# The complete mitochondrial genome of a blowfly *Calliphora nigribarbis* (Vollenhoven, 1863) (Insecta: Diptera: Calliphoridae)

**DOI:** 10.1080/23802359.2019.1629346

**Published:** 2019-07-11

**Authors:** Mustafa Zafer Karagozlu, Jung-Il Kim, Seong Hwan Park, Sang-Eon Shin, Chang-Bae Kim

**Affiliations:** a Department of Biotechnology, Sangmyung University, Seoul, Korea;; b Department of Legal Medicine, Korea University College of Medicine, Seoul, Korea

**Keywords:** Insecta, Diptera, Calliphoridae, complete mitochondrial genome, *Calliphora nigribarbis*

## Abstract

In the present study, the complete mitochondrial genome of a blowfly *Calliphora nigribarbis* has been sequenced and analyzed. The length of complete the mitochondrial genome is 16,279 bp, with 39.50% A, 13.20% C, 9.30% G, and 38.0% T nucleotide distribution. The complete mitochondrial genome consists of 13 protein-coding genes, 22 transfer RNAs, and 2 ribosomal RNAs likewise the most metazoan mitochondrial genomes. Furthermore, phylogenetic relationships of *C. nigribarbis* in the subfamily Calliphorinae investigated. The results suggested that *C. vomitoria* is the most related species to *C. nigribarbis* and the genus *Calliphora* is not monophyletic. This study provides the first complete mitochondrial genome sequence for the species.

*Calliphora nigribarbis* (=*Calliphora lata*) is a cryophilic blowfly distributed in East Asia (Peris and González-Mora 1989). They have a characteristic temperate zone life cycle, and it has been reported that their larval habitats are dung and carcass (Sawabe et al. 2009). According to these species characteristics, they are important model organisms for forensic studies (Komagata et al. 2006). In the present study, the complete mitochondrial genome of the *C. nigribarbis* has been sequenced and analyzed. This is the first complete mitochondrial genome record for the species and the fourth record for the genus.

The *C. nigribarbis* specimen was collected from the forest, Mt. Gaewun, Seoul/37°35′43.0″N 127°01′43.1″E, May 2016 and identified by DNA barcoding. The specimens deposited in the Department of Legal Medicine, Korea University (16La05). The complete mitochondrial genome was sequenced from the total DNA extracted from the legs and thorax. The methods for the complete mitochondrial genome sequencing and phylogenetic tree reconstruction were explained in our previous article (Karagozlu et al. 2016).

The length of complete mitochondrial genome is 16,279 bp, with the overall 77.50% AT content (GeneBank accession number MK893470). The nucleotide distribution for the mitochondrial genome is 39.50% A, 13.20% C, 9.30% G, and 38.0% T. The structure of the mitochondrial genome is typical in insect mitochondrial genome (Cameron 2014) which consists of 13 protein-coding genes, 22 transfer RNAs, and 2 ribosomal RNAs. Among these 37 genes, 23 genes encoded on the majority strand while remaining 14 genes encoded on the minority strand. There are three more complete mitochondrial genomes recorded belong to the genus *Calliphora* (*C. vicina, C. vomitoria,* and *C. chinghaiensis*) (Nelson et al. 2012; Chen et al. 2016; Ren et al. 2016). In comparison, *C. nigribarbis* has the longest complete mitochondrial genome. The size difference with the shortest record is 1100 bp (*C. chinghaiensis)*. The main reason for the size difference is the control region. The entire non-coding AT-rich region area between12S rRNA and tRNA-Ile is typically annotated as the ‘control region’ in the insect mitochondrial genome (Cameron 2014) and this area in the *C. nigribarbis* is 1460 bp in length which is the longest among four *Calliphora* records.

**Figure 1.**
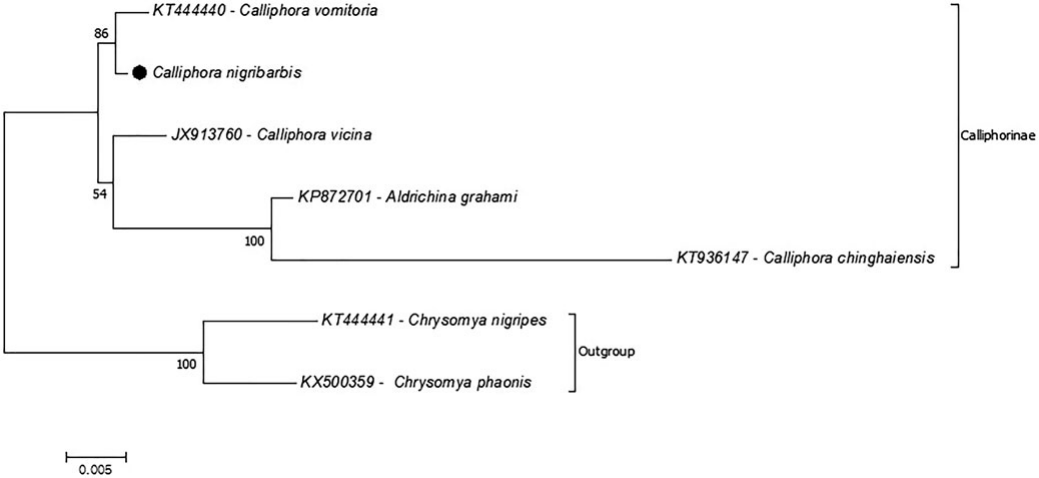
Phylogenic relationships of the Calliphora nigribarbis in the subfamily Calliphorinae. The phylogenetic tree was reconstructed by using the amino acid sequence of the 13 mitochondrial proteincoding genes. The two species from the subfamily Chrysomyinae represents outgroup. The complete mitochondrial genome data retrieved from GenBank.

Additionally, the phylogenic relationships of *C. nigribarbis* in the subfamily Calliphorinae were analyzed (Figure 1). For analysis, the phylogenetic tree was reconstructed by concatenated 13 protein-coding genes of complete mitochondrial genomes. *Calliphora* *vormitoria* is the most related species, and *C. chinghaiensis* is the least related species to *C. nigribarbis.* According to phylogenetic analysis, the genus *Calliphora* was not monophyletic in the subfamily Calliphorinae. *Calliphora chinghaiensis* clustered with a different genus species, *Aldrichina grahami*. However, as a result of limited complete mitochondrial genome records, the exact phylogenic relationship of the *Calliphora* species has not been confirmed. The previous studies based on one mitochondrial (COI), three nuclear (CPS, EF1a, and 28S ribosomal RNA) (Singh and Wells 2013), and 13 mitochondrial protein-coding genes (Chen et al. 2016) also showed the similar results. This study provides additional data for mitochondrial genome library the genus *Calliphora*.
